# The Onset and Progression of Hippocampal Synaptic Plasticity Deficits in the Q175FDN Mouse Model of Huntington Disease

**DOI:** 10.3389/fncel.2019.00326

**Published:** 2019-07-17

**Authors:** Jade G. Quirion, Matthew P. Parsons

**Affiliations:** Division of Biomedical Sciences, Faculty of Medicine, Memorial University of Newfoundland, St. John’s, NL, Canada

**Keywords:** long-term potentiation, Huntington disease, huntingtin, neurodegenerative disease, synaptic plasticity

## Abstract

Huntington disease (HD) is an inherited neurodegenerative disease characterized by a clinical triad of motor, psychiatric and cognitive symptoms. HD is caused by a CAG repeat expansion in the gene encoding the huntingtin protein. Homozygosity for the HD-causing mutation is extremely rare; thus, the majority of HD patients express the mutant huntingtin protein in addition to reduced levels of the non-pathogenic huntingtin protein. Deficits in synaptic plasticity, including hippocampal long-term potentiation (LTP), have been identified in various mouse models of HD and are thought to contribute to the debilitating cognitive symptoms associated with the disease. However, the bulk of these studies used N-terminal fragment or homozygous knock-in mouse models of HD at symptomatic ages, and our understanding of the onset and progression of synaptic plasticity deficits in the HD brain is lacking. To better understand the time-course of synaptic plasticity deficits in HD, as well as the impact of heterozygous and homozygous huntingtin mutations, we quantified basal synaptic connectivity, presynaptic release probability, presynaptically mediated post-tetanic potentiation (PTP) and postsynaptically mediated LTP at presymptomatic, early symptomatic and late symptomatic ages in heterozygous and homozygous Q175FDN knock-in HD mice. Our results demonstrate clear age-dependent effects of the HD-causing mutation on both short and long-term plasticity that generally emerge earlier in homozygous mice. Interestingly, deficits in presynaptic short-term plasticity were more closely linked to disease progression than deficits in postsynaptic LTP, and heterozygous mice were more susceptible to an LTP deficit when induced by high frequency stimulation compared to theta burst stimulation. To the best of our knowledge, the present study represents the most thorough characterization to date of the onset and progression of hippocampal synaptic plasticity deficits in a mouse model of HD, and should prove valuable to future studies exploring cellular mechanisms underlying the debilitating cognitive decline in HD.

## Introduction

Huntington disease (HD) is a fatal autosomal dominant neurodegenerative disease that affects approximately 1–2 individuals per 10,000 (Fisher and Hayden, 2014). HD is caused by an expansion of the CAG repeat in the first exon of the gene that encodes the huntingtin protein. The presence of the resultant mutant huntingtin protein, and perhaps in combination with reduced expression levels of non-expanded (i.e., non-pathogenic) huntingtin (Cattaneo et al., 2005; Dietrich et al., 2017), results in the progressive deterioration of much of the neuroaxis, with particularly devastating effects observed in the striatum. The symptoms of HD are described as a clinical triad that includes progressive motor and cognitive decline as well as psychiatric disturbances. The monogenic nature of HD has resulted in the identification of presymptomatic gene carriers (pre-HD); from this pre-HD population, it was discovered that subtle impairments in cognitive function are often evident as many as 15 years prior to a formal diagnosis based on unequivocal motor signs (Paulsen et al., 2008). In fact, in a population of pre-HD individuals predicted to be at least 14 years away from a formal diagnosis of HD, approximately 40% reached the criteria for mild cognitive impairment, with higher rates observed in those closer to a motor diagnosis (Duff et al., 2010). Deficits in episodic memory, processing speed, executive function and visuospatial perception were all found to be associated with pre-HD (Duff et al., 2010). While the bulk of preclinical HD research focuses on striatal dysfunction and motor impairment in HD animal models, it is well-accepted that extrastriatal dysfunction can make key contributions to the non-motor symptoms of HD (Ruocco et al., 2006; Ross et al., 2014). Of particular interest to the present study is HD-associated pathophysiology in the hippocampus, a brain region critically involved in cognitive function.

Prior to neurodegeneration, impairments in synaptic structure and function are readily detected in the striatum in HD mouse models (Milnerwood and Raymond, 2010; Raymond et al., 2011). Like Alzheimer disease (Selkoe, 2002), the early stages of HD have been associated with numerous “synaptic failures” (Selkoe, 2002) that are thought to promote subsequent neurodegeneration (Li et al., 2003; Okamoto et al., 2009; Milnerwood and Raymond, 2010; Milnerwood et al., 2010; Raymond et al., 2011; Parsons and Raymond, 2014). Various forms of synaptic plasticity, the cellular underpinnings of learning and memory, are impaired in mouse models of HD (Usdin et al., 1999; Murphy et al., 2000; Cummings et al., 2006, 2007; Milnerwood et al., 2006; Lynch et al., 2007; Simmons et al., 2009; Brito et al., 2014; Kolodziejczyk et al., 2014; Giralt et al., 2017; Sepers et al., 2017; Zhang et al., 2018). N-methyl-D-aspartate receptor (NMDAR)-dependent long-term potentiation (LTP) at the Schaffer collateral-CA1 synapse in the hippocampus is well-accepted to underlie cognitive processes such as learning and memory (Bliss and Collingridge, 1993; Bruel-Jungerman et al., 2007a,b; Citri and Malenka, 2008). While this particular form of LTP has previously been shown to be impaired in mouse models of HD, many of these studies were conducted at a symptomatic age in N-terminal fragment (Murphy et al., 2000; Anglada-Huguet et al., 2014; Giralt et al., 2017), overexpression (Hodgson et al., 1999) or homozygous knock-in HD mouse models (Lynch et al., 2007; Simmons et al., 2009); thus, while it is well-understood that HD is associated with poor synaptic plasticity, the onset and progression of plasticity impairments are poorly documented. As cognitive decline can emerge in the HD prodrome, it is important to understand what forms of synaptic plasticity, if any, are perturbed at presymptomatic ages in preclinical disease models. In the present study, we provide the most thorough characterization to date of hippocampal plasticity deficits in a mouse model of HD. Q175FDN mice express an expanded CAG tract in the gene encoding the huntingtin protein, and express higher levels of the mutant huntingtin protein than previous knock-in models of HD (Pouladi et al., 2013; Southwell et al., 2016). Altered electrophysiological properties, including reduced excitatory postsynaptic currents, have been detected in the striatum from both heterozygous and homozygous Q175FDN mice (Southwell et al., 2016); to the best of our knowledge, hippocampal synaptic plasticity has yet to be investigated in Q175FDN mice. We chose the novel Q175FDN knock-in mouse model for the present study due to its high expression level of mutant huntingtin and clear HD-like phenotype in both heterozygous and homozygous mice. Our results demonstrate clear deficits in both short- and long-term synaptic plasticity that exhibit differential sensitivities to disease progression, plasticity induction paradigm as well as mutant huntingtin expression levels and/or the loss of non-pathogenic huntingtin.

## Materials and Methods

### Animals

Male and female heterozygous and homozygous Q175FDN knock-in HD mice as well as wild type FVB/N littermates were used in the present study. Q175FDN breeders were obtained from the University of British Columbia and heterozygous × heterozygous breeding pairs were established at Memorial University of Newfoundland to produce a mix of heterozygous, homozygous, and wild-type (WT) littermates. Mice were group housed, exposed to a 12:12 light/dark cycle and standard chow and water were available *ad libitum*. Ear notching was performed, and tail samples were obtained prior to weaning for identification and genotyping purposes, respectively. For a subset of mice, purified DNA was sent to Laragen (Los Angeles, CA, United States) for CAG sizing. Mice with repeat lengths around 205 were selected as breeders to avoid spontaneous expansion of the CAG tract within the colony. All experiments with live animals were approved by the Memorial University’s Animal Care Committee in accordance with the guidelines set by the Canadian Council on Animal Care.

### Hippocampal Slice Preparation

With the exception of the slicing orientation, hippocampal slices were obtained as described previously (Pinky et al., 2018). Briefly, mice were deeply anesthetized with isoflurane and their brains were quickly extracted and submerged in ice-cold oxygenated slicing solution consisting of (in mM): 125 NaCl, 2.5 KCl, 25 NaHCO_3_, 1.25 NaH_2_PO_4_, 2.5 MgCl_2_, 0.5 CaCl_2_, 10 Glucose). The brain was blocked anterior and posterior to the hippocampus in the coronal plane and the two hemispheres were separated by a midsagittal cut. Each hemisphere was then further blocked by cutting the ventrolateral surface at and angle of approximately 20–30°. Each hemisphere was then glued on their blocked, ventrolateral surface, to the stage of a vibratome. This blocking strategy yields a good approximation of the transverse plane. Transverse slices of 350 μm thickness were cut in ice-cold oxygenated slicing solution using a Leica VT1000S vibratome. Once obtained, slices were transferred to room temperature oxygenated artificial cerebrospinal fluid (ACSF) containing (in mM): 125 NaCl, 2.5 KCl, 25 NaHCO_3_, 1.25 NaH_2_PO_4_, 1 MgCl_2_, 2 CaCl_2_, 10 Glucose. Slices were left to recover for a minimum of 90 min. After recovery, a single slice was transferred to a recording chamber, visualized under an Olympus BX-61 microscope and continuously perfused with ACSF saturated with 95%/5% O_2_/CO_2_. A peristaltic pump was used to maintain a flow rate between 1.5–2 ml/min and the ACSF temperature at the sample was held constant at 25°C using an in-line heater.

### Electrophysiology Recordings

Slices were visualized with a 4×/0.28 NA objective (Olympus) and an Andor iXon 897 camera. Micromanipulators were used to position glass stimulating and recording electrodes (resistance = 1.5–2.0 MΩ when filled with ACSF) in the hippocampus. The recording electrode was typically placed approximately 400 μm distal to the stimulating electrode, and both electrodes were positioned approximately 200 μm ventral to the cell body layer. Field excitatory postsynaptic potentials (fEPSP) were induced with single pulses (0.1 ms pulse width) using a stimulus intensity that elicits 30–40% of the maximum fEPSP response as determined from an input/output curve. Paired-pulse ratios were examined at inter-pulse intervals of 50, 100, and 250 ms. For PTP and LTP experiments, single pulses were administered every 20 s and a baseline was recorded until it was stable for at least 15 min. To be considered stable, baseline responses had to exhibit a non-significant regression line over the 15-min baseline period. To induce PTP/LTP, a theta burst stimulation (TBS) protocol was used which involves 10 bursts of 4 pulses each with a 200-ms delay between each burst. The 4 pulses within each burst were delivered at 100 Hz. Where noted, some slices were subjected to high frequency stimulation (HFS) instead of TBS. HFS consisted of a single train of 100 pulses at 100 Hz. Following TBS or HFS, fEPSP responses to single stimuli were recorded every 20 s for an additional 60 min.

### Electrophysiology Analysis

The fEPSP slopes for the input/output curve, paired-pulses, baseline and potentiation were analyzed using Clampfit 10 software. The slope was measured from the first 1.5–2 ms of the fEPSP. Percent LTP was calculated by averaging the response slopes for the last 5 min of recording after LTP induction (55–60 min post-induction) and expressed as the percent of the baseline average. PTP was calculated by averaging the response slopes for the first 3 min of recording immediately after LTP induction and expressed as a percentage of the baseline average. For burst analysis, the area under the curve of the fEPSP response to each of the ten bursts associated with TBS was calculated in Clampfit 10. The area of bursts 2–10 were then each expressed as the fold change of the area of the first burst.

### Estrous Cycle Staging

For female mice, estrous cycle staging was determined using vaginal lavage followed by crystal violet staining as described previously (McLean et al., 2012). LTP has been shown to be enhanced during the proestrus stage of the estrous cycle (Ooishi et al., 2012); thus, it was of interest to determine the cycle stage for each of the females in the present study at the time of dissection. Briefly, the opening of the vaginal canal was rinsed with distilled water and a pipette was used to expel and aspirate water 4–5 times in the opening of the vaginal canal. The fluid was then placed on a glass slide and allowed to dry at room temperature. Once dry, the slide was placed in crystal violet for 1 min followed by two washes in distilled water for 1 min. The slide was then coverslipped and examined using a light microscope. The ratio of cornified squamous epithelial cells, leukocytes, and/or nucleated epithelial cells was used to determine the estrous stage the mouse was in at the time of brain extraction as previously described (McLean et al., 2012). In the present study, we found no difference between measures of LTP (proestrus %LTP = 96.9 ± 27.7, *n* = 6; non-proestrus %LTP = 77.37 ± 9.51, *n* = 11, *t*-test *p* = 0.425) or PTP (proestrus %PTP = 158.3 ± 41.5, *n* = 6; non-proestrus %PTP = 179.2 ± 22.0, *n* = 11, *t*-test *p* = 0.629) when comparing WT females in proestrus with those either estrus, metestrus or diestrus; therefore, the data were pooled.

### Statistical Analysis

GraphPad Prism 8 was used to conduct unpaired two-tailed *t*-tests, linear regression, one-way and two-way ANOVAs. Outliers were removed only if they fell outside three-fold of the standard deviation from the mean. Tukey tests were used for one-way ANOVA *post hoc* comparisons when a main effect was found. Bonferroni tests were used for two-way ANOVA *post hoc* comparisons when a main effect was found. *P*-values less than 0.05 were considered significant. All error bars represent standard error of the mean (SEM) and reported n-values refer to the number of experiments (slices) obtained from a minimum of four animals. The statistical test used for each experiment is reported in the results text.

## Results

Here, we sought to thoroughly characterize the onset and progression of synaptic plasticity deficits in a slowly progressing mouse model of HD. We focused on the novel Q175FDN HD model, a knock-in model in which a clear HD-like phenotype begins to develop around 6–9 months of age in heterozygous mice due to high expression levels of the mutant huntingtin protein. In Q175FDN homozygous mice, which completely lack non-pathogenic huntingtin and are therefore less relevant to the large majority of human HD patients, the HD-like phenotype appears earlier and is more severe compared to that observed in Q175FDN heterozygous mice (Southwell et al., 2016). In the present study, experiments were performed at different ages and data were separated into the following three age groups: 3 months (101.5 ± 1.8 days), 6–9 months (215.8 ± 8.4 days), and 12 months (367.9 ± 2.4 days). For Q175FDN heterozygous mice, these ages represent presymptomatic, early symptomatic and late symptomatic disease stages, respectively, while the HD-like phenotype begins to emerge in Q175FDN homozygous mice as early as 3 months (Southwell et al., 2016).

**FIGURE 1:**
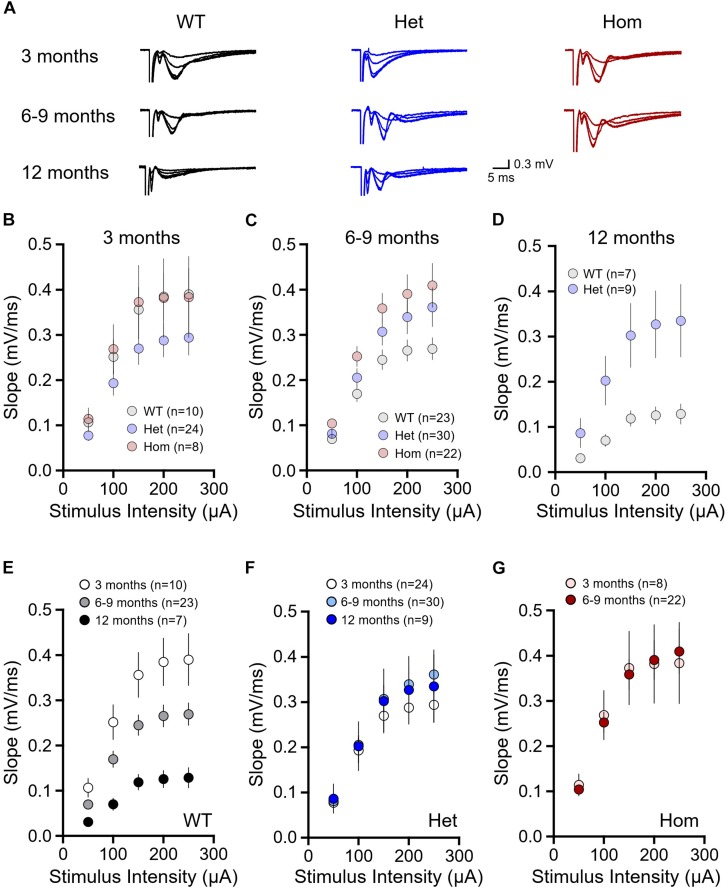
Basal synaptic strength is higher in Q175FDN mice at early- and late-symptomatic disease stages. **(A)** Representative field potential traces recorded from CA1 stratum radiatum in response to single pulse Schaffer collateral stimulation. Shown, increasing in size, are responses to stimulation intensities of 50, 100, 150, 200, and 300 μA from WT (black), heterozygous Q175FDN (Het; blue) and homozygous Q175FDN (Hom; red) mice. Scale bars apply to all traces. **(B)** fEPSP responses to increasing stimulus intensities in 3-month-old WT, Het Q175FDN and Hom Q175FDN mice. **(C)** fEPSP responses to increasing stimulus intensities in 6- to 9-month-old WT, Het Q175FDN and Hom Q175FDN mice. **(D)** fEPSP responses to increasing stimulus intensities in 12-month-old WT and Het Q175FDN mice. **(E)** fEPSP responses to increasing stimulus intensities in 3, 6–9, and 12-month-old WT mice. **(F)** fEPSP responses to increasing stimulus intensities in 3, 6–9, and 12-month-old Het Q175FDN mice. **(G)** fEPSP responses to increasing stimulus intensities in 3 and 6- to 9-month-old Hom Q175FDN mice. Error bars represent mean ± SEM.

First, we examined basal synaptic connectivity at the Schaffer collateral-CA1 synapse in acute brain slices obtained from WT, Q175FDN heterozygous and Q175FDN homozygous mice at 3, 6–9 or 12 months of age. At 3 months, input–output curves revealed that the slope of the fEPSP response increased to stimulus pulses of increasing intensity as expected, but to the same extent in all mice and no significant genotype or interaction effects were observed (Figures 1A,B, WT *n* = 10, heterozygous *n* = 24, homozygous *n* = 8; repeated-measures two-way ANOVA, *p*_(genotype)_ = 0.280, *p*_(intensity)_ < 0.001, *p*_(interaction)_ = 0.484). At 6–9 months, we found that at higher stimulus intensities, mean fEPSP slopes were elevated in heterozygous and homozygous mice compared to WTs, and a significant interaction effect was observed (Figures 1A,C, WT *n* = 23, heterozygous *n* = 30, homozygous *n* = 22; repeated-measures two-way ANOVA, *p*_(genotype)_ = 0.051, *p*_(intensity)_ < 0.001, *p*_(interaction)_ = 0.031). At 12 months, we also observed larger fEPSP basal responses in heterozygous mice compared to WT mice (Figures 1A,D, WT *n* = 7, heterozygous *n* = 9, repeated-measures two-way ANOVA, *p*_(genotype)_ = 0.047, *p*_(intensity)_ < 0.001, *p*_(interaction)_ < 0.001). Homozygous mice were not included in this 12 month cohort, as their survival curves begin to drop sharply after approximately 9 months of age (Southwell et al., 2016).

As we observed significant interaction and genotype effects in the older cohorts, we sought to determine how age affects fEPSP responses within a given genotype. WT fEPSP response slopes significantly diminished with age (Figures 1A,E, 3 months *n* = 10, 6–9 months *n* = 23, 12 months *n* = 7, repeated-measures two-way ANOVA, *p*_(age)_ < 0.001, *p*_(intensity)_ < 0.001, *p*_(interaction)_ < 0.001), in agreement with a previous study on the effects of age on Schaffer collateral-CA1 basal connectivity (Weber et al., 2015). Surprisingly, the effect of age on fEPSP slope was not observed in either heterozygous (Figures 1A,F, 3 months *n* = 24, 6–9 months *n* = 30, 12 months *n* = 9, repeated-measures two-way ANOVA, *p*_(age)_ = 0.711, *p*_(intensity)_ < 0.001, *p*_(interaction)_ = 0.534) or homozygous mice (Figures 1A,G, 3 months *n* = 8, 6–9 months *n* = 22, repeated-measures two-way ANOVA, *p*_(age)_ = 0.984, *p*_(intensity)_ < 0.001, *p*_(interaction)_ = 0.890). Therefore, the significant differences observed between genotypes appears to result from an abnormal maintenance of strong basal connectivity with age in heterozygous and homozygous mice that is not seen in WT mice.

**FIGURE 2:**
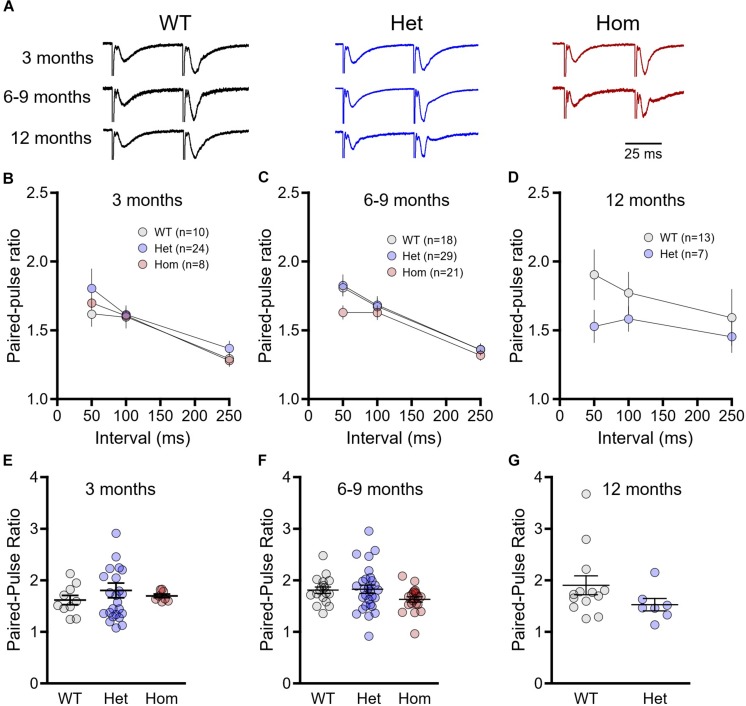
Paired-pulses ratios are not significantly different between wild-type (WT) and Q175FDN mice. **(A)** Representative field potential traces recorded from CA1 stratum radiatum in response to single pulse Schaffer collateral stimulation. Shown are responses to paired pulses (50 ms inter-pulse interval) from wild-type (WT; black), heterozygous Q175FDN (Het; blue) and homozygous Q175FDN (Hom; red) mice. Trace heights were scaled to match the size of the first response. Horizontal scale bar applies to all traces. **(B)** Paired-pulse ratios for interpulse-intervals of 50, 100 and 250 ms in 3-month-old WT (gray), heterozygous Q175FDN (Het; blue) and homozygous Q175FDN (Hom; red) mice. **(C)** Paired-pulse ratios for interpulse-intervals of 50, 100 and 250 ms in 6- to 9-month-old WT, Het Q175FDN and Hom Q175FDN mice. **(D)** Paired-pulse ratios for interpulse-intervals of 50, 100 and 250 ms in 12-month-old WT and Het Q175FDN mice. **(E)** Paired-pulse ratios for 50 ms interpulse-intervals in 3-month-old WT, Het Q175FDN and Hom Q175FDN mice. **(F)** Paired-pulse ratios for 50 ms interpulse-intervals in 6- to 9-month-old WT, Het Q175FDN and Hom Q175FDN mice. **(G)** Paired-pulse ratios for 50 ms interpulse-intervals in 12-month-old WT and Het Q175FDN mice. Data are presented as mean ± SEM for panels **(B–D)**. Individual data points as well as mean ± SEM shown in panel **(E–G)**.

Next, we assessed presynaptic release probability through standard paired-pulse ratio measurements (Figure 2A). At 3 months of age, paired-pulse ratios decreased with increasing inter-pulse intervals as expected, although no genotype differences were observed (Figure 2B, WT *n* = 10, heterozygous *n* = 24, homozygous *n* = 8, repeated-measures two-way ANOVA, *p*_(genotype)_ = 0.280, *p*_(interval)_ < 0.001, *p*_(interaction)_ = 0.484). Similar results showing no significant genotype or interaction effects were obtained for the 6–9 month cohort (Figure 2C, WT *n* = 18, heterozygous *n* = 29, homozygous *n* = 21, repeated-measures two-way ANOVA, *p*_(genotype)_ = 0.227, *p*_(interval)_ < 0.001, *p*_(interaction)_ = 0.441) and the 12 month cohort (Figure 2D, WT *n* = 13, heterozygous *n* = 7, repeated-measures two-way ANOVA, *p*_(genotype)_ = 0.281, *p*_(interval)_ = 0.339, *p*_(interaction)_ = 0.664). As previous research demonstrates a significant paired-pulse ratio difference at short intervals in HD mice (Kolodziejczyk et al., 2014), we isolated the 50 ms interval data and compared the genotypes within each age group with a one-way ANOVA. There were no genotype differences at 3 months of age (Figure 2E, WT *n* = 10, heterozygous *n* = 24, homozygous *n* = 8, one-way ANOVA, *p* = 0.657), and while mean paired-pulse ratios tended to be lower in homozygous mice at 6–9 months (Figure 2F, WT *n* = 18, heterozygous *n* = 29, homozygous *n* = 21, one-way ANOVA, *p* = 0.107) and in heterozygous mice at 12 months (Figure 2G, WT *n* = 13, heterozygous *n* = 7, unpaired *t*-test, *p* = 0.178), these results were not statistically significant. Thus, we conclude that at the Shaffer collateral-CA1 synapse, presynaptic release probability is minimally affected, if at all, in both pre- and postsymptomatic ages in heterozygous and homozygous Q175FDN mice.

**FIGURE 3:**
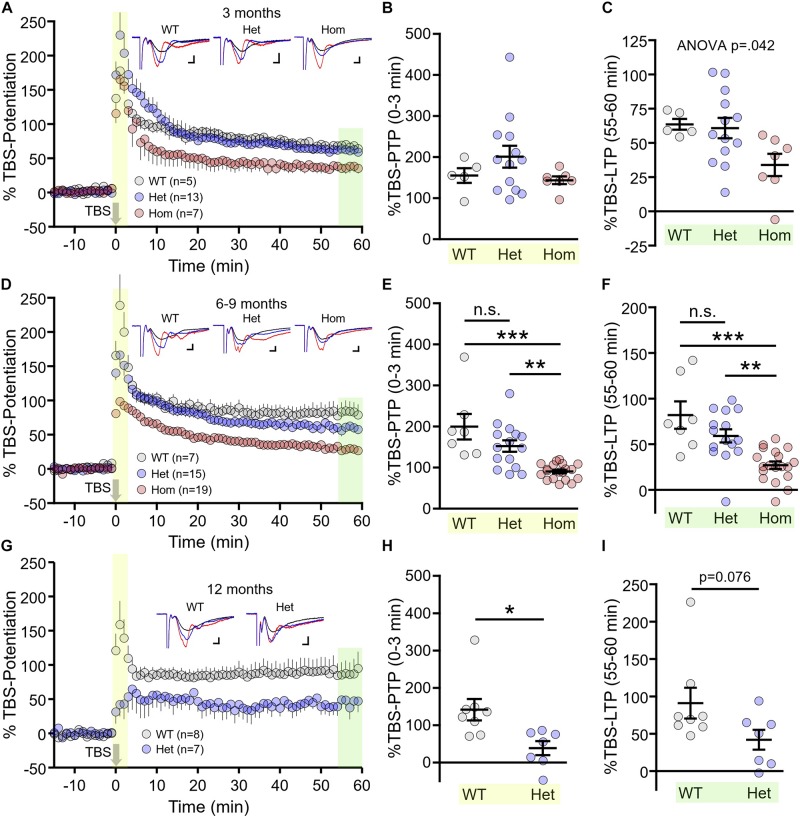
Short- and long-term synaptic plasticity deficits in Q175FDN mice. **(A)** Graph of percent potentiation for 1 h following theta-burst stimulation (TBS; applied at time = 0) in hippocampal tissue from 3-month-old wild-type (WT; gray), heterozygous Q175FDN (Het; blue) and homozygous Q175FDN (Hom; red) mice. Yellow shading indicates the responses that were averaged to quantify post-tetanic potentiation (PTP). Green shading indicates the responses that were averaged to quantify long-term potentiation (LTP). **(B)** Individual data points (circles) and mean ± SEM (black lines) of the percent PTP in 3-month-old WT, Het Q175FDN and Hom Q175FDN mice. **(C)** Individual data points (circles) and mean ± SEM (black lines) of the percent LTP in 3-month-old WT, Het Q175FDN and Hom Q175FDN mice. Panels **(D–F)** same as panels **(A–C)** but in mice aged 6–9 months. Panels **(G–I)** same as panel **(A–C)** but in mice aged 12 months and restricted to WT and Het Q175FDN mice. Representative traces in panels **(A)**, **(D)**, and **(G)** represent traces obtained during baseline (black), PTP (red), and LTP (blue) for the indicated genotype. Scale bars for the representative traces indicate 2 ms and 0.2 mV. ^*^p < 0.05, ^∗∗^*p* < 0.01, ^∗∗∗^*p* < 0.001.

Next, we assessed another form of presynaptic short-term plasticity, PTP, as well as long-term postsynaptic plasticity at Shaffer collateral-CA1 synapses in WT, heterozygous and homozygous Q175FDN mice at the aforementioned age groups. In these experiments, plasticity was induced by TBS consisting of 10 bursts of 4 pulses separated by an inter-burst interval of 200 ms. At 3 months, we found no significant difference in PTP induced by TBS (herein referred to as TBS–PTP), quantified by averaging the fEPSP slope for the first 3 min after TBS and expressing this average as a percentage of the baseline fEPSP slope prior to TBS (Figures 3A,B, WT *n* = 5, heterozygous *n* = 13, homozygous *n* = 7, one-way ANOVA, *p* = 0.220). While short-term PTP was intact, we found that LTP induced by TBS (herein referred to as TBS–LTP), quantified 55–60 min post-TBS, was highly variable in heterozygous mice while mean responses tended to be lower in homozygous mice. At this age, we observed a significant effect of genotype on LTP magnitude (Figures 3A,C, WT *n* = 5, heterozygous *n* = 13, homozygous *n* = 7, one-way ANOVA, *p* = 0.042), although no significance difference between any two particular genotypes was detected with *post hoc* comparisons (Tukey *post hoc p* > 0.05 for all comparisons).

At 6–9 months, TBS–PTP was significantly affected by genotype (Figures 3D,E, WT *n* = 7, heterozygous *n* = 15, homozygous *n* = 19, *p* < 0.001). Mean TBS–PTP values were highest for WT mice and lowest for homozygous mice, with intermediate levels observed in heterozygous mice (Tukey *post hoc*: WT vs. heterozygous *p* > 0.05; WT vs. homozygous *p* < 0.001; heterozygous vs. homozygous *p* < 0.01). A similar pattern was observed for TBS–LTP, with LTP strength being highest in WT mice, lowest in homozygous Q175FDN mice and intermediate in heterozygous Q175FDN mice (Figures 3D,F, WT *n* = 7, heterozygous *n* = 15, homozygous *n* = 19, one-way ANOVA, *p* < 0.001, Tukey *post hoc*: WT vs. heterozygous *p* > 0.05; WT vs. homozygous *p* < 0.001; heterozygous vs. homozygous *p* < 0.01). These data clearly demonstrate a greater impairment of both short- and long-term plasticity in homozygous mice compared to heterozygous mice.

As before, only WT and heterozygous mice were tested at 12 months of age, as a result of the short lifespan in homozygous mice (Southwell et al., 2016). PTP was significantly impaired in 12 month old heterozygous mice compared to age-matched WT littermates (Figures 3G,H, WT *n* = 8, heterozygous *n* = 7, unpaired *t*-test, *p* = 0.012). While LTP tended to be reduced in 12 month HETs, this result did not quite reach statistical significance (Figures 3G,I, WT *n* = 8, heterozygous *n* = 7, unpaired *t*-test, *p* = 0.076).

**FIGURE 4:**
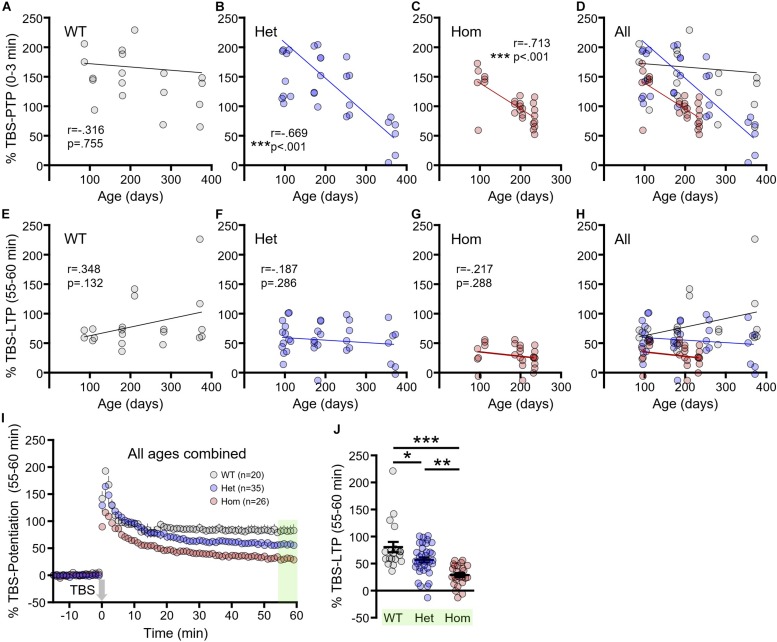
The progression of short- and long-term synaptic plasticity deficits in Q175FDN mice. **(A)** Linear regression showing no significant relationship between post-tetanic potentiation (PTP) and age in wild-type (WT) mice. **(B)** Linear regression showing a significant negative relationship between PTP and age in heterozygous Q175FDN (Het) mice. **(C)** Linear regression showing a significant negative relationship between PTP and age in homozygous Q175FDN (Hom) mice. **(D)** Combined graph from the data in panels **(A–C)**. **(E–G)** Linear regression showing no significant relationship between long-term potentiation (LTP) and age in WT **(E)**, Het **(F)** and Hom **(G)** mice. **(H)** Combined graph from the data in panels **(E–G)**. **(I)** Graph of percent potentiation for 1 h following theta-burst stimulation (TBS; applied at time = 0) in hippocampal tissue from WT, Het and Hom mice of all ages. Green shading indicates the responses that were averaged to quantify LTP. **(J)** Individual data points (circles) and mean ± SEM (black lines) of the percent LTP in WT, Het and Hom mice of all ages. ^*^p < 0.05, ^∗∗^*p* < 0.01, ^∗∗∗^*p* < 0.001.

To further clarify how disease progression affects short- and long-term synaptic plasticity, we correlated the magnitude of TBS–PTP and TBS–LTP with age (in days) for each genotype. TBS–PTP was not significantly affected by age in WT mice (Figure 4A, *n* = 20, *r* = −0.316, *p* = 0.755). In contrast, a significant negative correlation was observed between TBS–PTP and age for both heterozygous (Figure 4B, *n* = 35, *r* = −0.669, *p* < 0.001) and homozygous mice (Figure 4C, *n* = 26, *r* = −0.713, *p* < 0.001). That is, TBS–PTP significantly decreased with disease progression in both heterozygous and homozygous mice (Figure 4D). Surprisingly, and in contrast to TBS–PTP, TBS–LTP was not significantly affected by age in WT (Figure 4E, *n* = 20, *r* = 0.348, *p* = 0.132), heterozygous (Figure 4F, *n* = 35, *r* = −0.187, *p* = 0.286) or homozygous mice (Figure 4G, *n* = 26, *r* = −0.217, *p* = 0.288). These data demonstrate that deficits in presynaptic short-term plasticity are more closely linked to disease progression than deficits in postsynaptic long-term plasticity. As age had no significant effect on TBS–LTP measures in either genotype, we pooled the data from all ages (Figures 4I,J). Note that the data presented in Figures 4I,J represent the combined data from Figures 3A,D,G. By combining the three different age groups, the higher n-values now revealed significant *post hoc* LTP deficits for both heterozygous and homozygous mice, with the deficit being more pronounced in the latter (Figures 4I,J, WT *n* = 20, heterozygous *n* = 35, homozygous *n* = 26, one way-ANOVA, *p* < 0.001, Tukey *post hoc*: WT vs. heterozygous *p* < 0.05; WT vs. homozygous *p* < 0.001; heterozygous vs. homozygous *p* < 0.01). In all, these results suggest that while deficits in both short- and long-term plasticity exist in the HD hippocampus, short-term plasticity deficits may be more closely linked to disease progression than long-term plasticity deficits.

**FIGURE 5:**
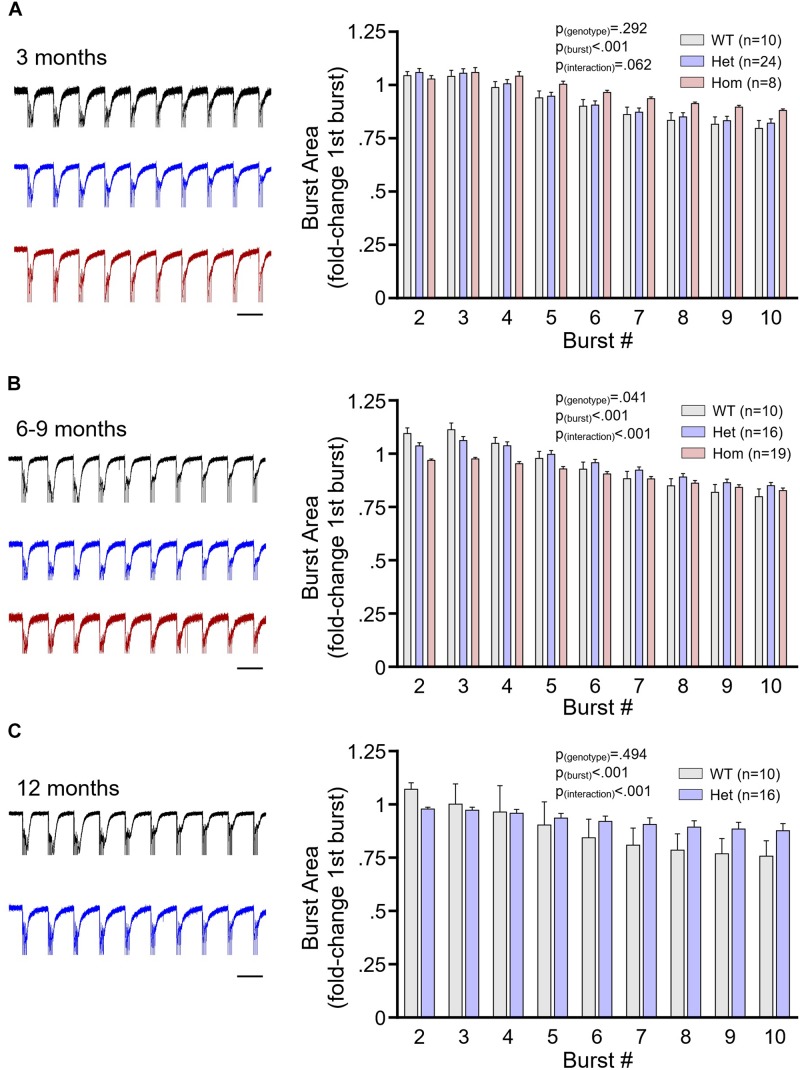
The postsynaptic population response to plasticity-inducing stimulation. **(A)** Mean ± SEM responses of the area under the curve of the CA1 stratum radiatum field potential response to theta burst stimulation (TBS) of the Schaffer collateral pathway. Data are normalized to the area under the curve of the response to the first burst of TBS. Data shown are from 3-month-old wild-type (WT; gray), heterozygous Q175FDN (Het; blue) and homozygous Q175FDN (Hom; red) mice. Panel **(B)** same as panel **(A)** but for WT, Het and Hom mice aged 6–9 months. Panel **(C)** same as panel **(A)** but for WT and Het mice aged 12 months. Representative fEPSP responses to TBS are shown on the left. Scale bars = 200 ms. Traces are scaled to match the size of the response to the first burst TBS. Black trace = WT; blue trace = Het; red trace = Hom.

It was previously shown that symptomatic homozygous knock-in *Hdh*^*Q111*^ mice exhibit a reduced CA1 stratum radiatum population response to TBS of the Schaffer collateral pathway (Lynch et al., 2007). Thus, we were interested in determining whether Q175FDN mice exhibited abnormal population responses during the duration of the TBS itself. We analyzed the area under the curve of the fEPSP response to each burst of the TBS in WT as well as heterozygous and homozygous Q175FDN mice at the aforementioned presymptomatic, early symptomatic and late symptomatic ages. At 3 months, the fEPSP response to TBS was not significantly different between WT, heterozygous and homozygous mice, and no significant genotype or interaction effects were observed (Figure 5A, WT *n* = 10, heterozygous *n* = 24, homozygous *n* = 8, repeated-measures two-way ANOVA, *p*_(genotype)_ = 0.292, *p*_(burst)_ < 0.001, *p*_(interaction)_ = 0.062). The trending interaction effect largely reflects the non-significant tendency for homozygous mice to maintain larger mean fEPSP responses throughout the duration of the TBS. In contrast, at 6–9 months, heterozygous and homozygous mice exhibited lower mean fEPSP responses early in the TBS, and significant genotype and interaction effects were observed (Figure 5B, WT *n* = 10, heterozygous *n* = 16, homozygous *n* = 19, repeated-measures two-way ANOVA, *p*_(genotype)_ = 0.041, *p*_(burst)_ < 0.001, *p*_(interaction)_ < 0.001; Bonferroni *post hoc*: *p* > 0.05 for all comparisons). At 12 months, heterozygous responses to TBS tended to be lower than WT responses early in TBS but exhibited less desensitization throughout; this pattern resulted in a significant interaction effect in the absence of a genotype effect (Figure 5C, WT *n* = 7, heterozygous *n* = 8, repeated-measures two-way ANOVA, *p*_(genotype)_ = 0.494, *p*_(burst)_ < 0.001, *p*_(interaction)_ < 0.001; Bonferroni *post hoc*: *p* > 0.05 for all comparisons).

**FIGURE 6:**
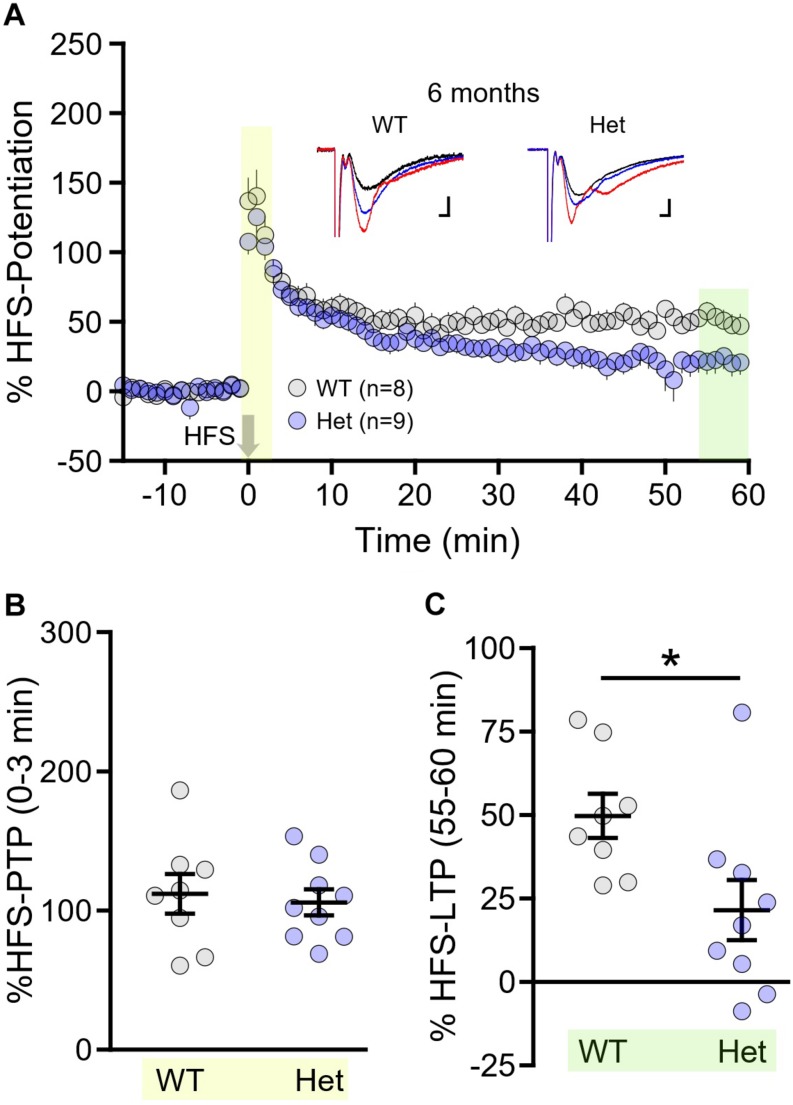
Long-term potentiation (LTP) induced by high-frequency stimulation (HFS) is impaired in Q175FDN mice as early as 6 months of age. **(A)** Graph of percent potentiation for 1 h following HFS (applied at time = 0) in hippocampal tissue from wild-type (WT; gray) and heterozygous Q175FDN mice (Het; blue) at 6 months of age. Yellow shading indicates the responses that were averaged to quantify post-tetanic potentiation (PTP). Green shading indicates the responses that were averaged to quantify LTP. **(B)** Individual data points (circles) and mean ± SEM (black lines) of the percent PTP in 6-month-old WT and Het mice. **(C)** Individual data points (circles) and mean ± SEM (black lines) of the percent LTP in 6-month-old WT and Het mice. Representative traces in panel **(A)** represent traces obtained during baseline (black), PTP (red) and LTP (blue) for the indicated genotype. Scale bars for the representative traces indicate 2 ms and 0.2 mV. ^*^p < 0.05.

While less physiologically relevant than TBS, high-frequency stimulation (HFS; 100 Hz, 1 s) is the most common stimulation paradigm used to evoke LTP. Different patterns of plasticity-inducing stimuli, including TBS and HFS, are known to produce LTP via distinct mechanisms (Zhu et al., 2015). As the mechanisms underlying the plasticity deficit in HD are not well-understood, any different sensitivities to one form of LTP over the other may help reveal abnormal signaling pathways in the HD hippocampus. Lastly, we sought to determine whether HFS–LTP was more or less susceptible to the HD-causing mutation compared to TBS–LTP. To address this question, we focus our efforts on WT and the more relevant heterozygous mice, and chose to conduct these experiments at 6 month of age (±2 weeks). At this age, and including mice up to 9 months of age, no significant TBS–LTP deficit was observed in heterozygous mice; homozygosity for the HD-causing mutation was required to observe a TBS–LTP deficit at this age. Interestingly, while 6 month heterozygous mice exhibited perfectly normal PTP following HFS (HFS–PTP, Figures 6A,B, WT *n* = 8, heterozygous *n* = 9, unpaired *t*-test, *p* = 0.715), a significant impairment of HFS–LTP was observed (Figure 6C, WT *n* = 8, heterozygous *n* = 9, unpaired *t*-test, *p* = 0.023). This result suggests that pathways required for HFS–LTP may be affected earlier in HD than those required for TBS–LTP, and also demonstrates that a clear LTP impairment can be observed in HD mice in the absence of a PTP deficit.

## Discussion

Hippocampal LTP deficits have been previously described in numerous HD mouse models (Hodgson et al., 1999; Murphy et al., 2000; Lynch et al., 2007; Simmons et al., 2009; Anglada-Huguet et al., 2014; Brito et al., 2014; Kolodziejczyk et al., 2014; Giralt et al., 2017; Zhang et al., 2018). Many of these studies were conducted at a symptomatic age in N-terminal fragment, overexpression or homozygous knock-in HD mouse models; thus, while it is well-understood that HD is associated with poor synaptic plasticity, the onset and progression of short- and long-term plasticity impairments are poorly documented. The present study extends on the existing literature by quantifying basal connectivity, presynaptic release probability as well as short-term presynaptic and long-term postsynaptic plasticity effects at three different disease stages in both heterozygous and homozygous knock-in HD mice. In addition, we quantified activity-dependent long-term plasticity in response to two commonly used LTP induction paradigms that are known to rely on different mechanisms (Zhu et al., 2015). To the best of our knowledge, the current study represents the most thorough characterization of the onset and progression of hippocampal plasticity deficits in a mouse model of HD to date.

### Basal Connectivity and Presynaptic Release Probability

An early study at the CA3-CA1 synapse found no basal difference in synaptic strength between WT mice and a knock-in mouse model of HD (Usdin et al., 1999). More recently, basal CA3-CA1 connectivity was shown to be enhanced in the aggressive R6/2 model of HD (Beaumont et al., 2014). Such discrepancies are likely due to the different models used and the different ages tested. In the present study, we compared basal CA3-CA1 connectivity in heterozygous and homozygous Q175FDN mice and their WT littermates at different stages of disease progression. By monitoring fEPSP responses to increasing stimulus intensities, we found that HD mice indeed had larger population responses as the disease progressed. Interestingly, the steeper fEPSP slopes observed in HD mice was not due to an age-dependent synaptic strengthening in these mice but was rather due to the tendency for responses to decrease with age in WT mice, a phenomenon that has been observed previously (Weber et al., 2015). This age-related decline in synapse strength was not observed in either heterozygous or homozygous Q175FDN mice. It is unlikely that the different synaptic responses to Schaffer collateral stimulation is due to alterations in presynaptic release probability, as no significant genotype differences were observed in paired-pulse ratios at any of the ages tested. It should be noted that our paired-pulse ratio results contrast with those from a few prior studies in other HD models which have shown reduced paired-pulse ratios (indicative of a higher initial release probability) at the CA3-CA1 synapse (Usdin et al., 1999; Kolodziejczyk et al., 2014). While our data trended towards a lower paired-pulse ratio for shorter intervals at 6–9 months (homozygous) and 12 months (heterozygous), the data did not reach statistical significance. It is entirely possible that a shorter interval (e.g., 20 ms) may have revealed a genotype difference in the present study; although prior research that has identified paired-pulse ratio differences in HD mice detected significant effects at intervals of 50 ms or longer (Usdin et al., 1999; Kolodziejczyk et al., 2014).

### Synaptic Plasticity Impairments in HD: Mutant Huntingtin Gain of Function or Non-pathogenic Huntingtin Loss of Function?

Homozygous knock-in mice completely lack non-pathogenic huntingtin and therefore do not recapitulate the balance of huntingtin and mutant huntingtin expression in the vast majority of HD patients. Prior studies have shown severe plasticity deficits in TBS–LTP in homozygous knock-in HD mice (Lynch et al., 2007; Simmons et al., 2009), although their heterozygous counterparts were not evaluated in these studies. Similarly, clear LTP deficits have been observed in N-terminal fragment R6/1 (Giralt et al., 2017) and R6/2 (Murphy et al., 2000) models, although these experiments were performed only at a symptomatic age. In the present study, we demonstrate that the TBS–PTP and TBS–LTP deficits in heterozygous Q175FDN mice, while apparent, are mild in comparison or occur much later to those observed in Q175FDN homozygous mice. Deleting the neomycin resistance cassette upstream of the huntingtin gene in Q175 mice resulted in a two-fold increase in mutant huntingtin protein expression in Q175FDN mice. Western blot quantification from cortical tissue demonstrated that heterozygous Q175FDN mice express mutant huntingtin at a level that is approximately 55% of the average expression of non-pathogenic huntingtin. That is, they produce half as much mutant huntingtin as they do non-pathogenic huntingtin (Southwell et al., 2016). In contrast, Q175FDN homozygous mice produce no non-pathogenic huntingtin and approximately twice the amount of mutant huntingtin than their heterozygous littermates. Thus, the greater/accelerated impairment of TBS–PTP and TBS–LTP in homozygous mice could be explained either by their lack of non-pathogenic huntingtin and/or elevated expression of mutant huntingtin. Indeed, it has been shown that non-pathogenic huntingtin promotes not only brain-derived neurotrophic factor (BDNF) production but also its axonal transport along microtubules as well as the dendritic retrograde transport of BDNF TrkB receptors (Zuccato et al., 2001; Gauthier et al., 2004; Liot et al., 2013). Both BDNF and its receptor TrkB play well-established roles in synaptic plasticity (Bramham and Messaoudi, 2005; Minichiello, 2009; Panja and Bramham, 2014). For example, it was recently demonstrated that an LTP-inducing stimulus resulted in rapid and sustained NMDAR-dependent TrkB receptor activation by postsynaptic BDNF, and that this autocrine signaling is essential to structural and functional LTP (Harward et al., 2016). Interestingly, exogenous BDNF application or treatment with an ampakine that increases BDNF expression is sufficient to restore robust LTP in homozygous knock-in HD mice (Lynch et al., 2007; Simmons et al., 2009). Similarly, treatment with tianeptine, an antidepressant reported to increase BDNF expression (Reagan et al., 2007), had a beneficial effect on AMPA receptor trafficking and LTP in numerous mouse models of HD (Zhang et al., 2018). One important question that remains unaddressed is whether the LTP deficit common to so many HD models is due to a toxic gain of function of mutant huntingtin, the loss of function of non-pathogenic huntingtin and/or the presence of mutant huntingtin interfering with the normal function of non-pathogenic huntingtin. In fact, abnormal synaptic plasticity is observed in many regions throughout the HD brain, not just the hippocampus (Smith-Dijak et al., 2019); the relative contributions of non-pathogenic huntingtin loss of function and mutant huntingtin gain of function to plasticity impairments throughout the HD brain is poorly understood. It is important for future studies to address this question directly as huntingtin-lowering agents, some of which are non-selective and will simultaneously reduce both non-pathogenic huntingtin and mutant huntingtin, enter the clinic (Wild and Tabrizi, 2017).

### Putative Mechanisms Underlying the Plasticity Deficits in HD Mice

In the present study, we demonstrated clear deficits in both PTP and LTP in heterozygous and homozygous Q175FDN mice. While both are indicative of a plasticity deficit in HD, it is worth emphasizing that PTP and LTP represent two very distinct forms of synaptic plasticity that rely on different mechanisms. It has been shown that PTP largely reflects a transient increase in presynaptic release probability in response to plasticity-inducing stimuli including TBS and HFS. PTP is thought to rely on presynaptic protein kinase C (PKC) and is associated with an enhanced calcium sensitivity of vesicle fusion events (Brager et al., 2003; Korogod et al., 2007), although this precise mechanism has been recently challenged (Wang et al., 2016). On the other hand, LTP induced by TBS or HFS at CA3-CA1 synapses is well known to be dependent on postsynaptic NMDAR activation. While both TBS–LTP and HFS–LTP are NMDAR-dependent, it has become clear that distinct postsynaptic signaling pathways are recruited following these different activity patterns. For example, consolidation of TBS–LTP relies more on calpain-1 and extracellular regulated kinase than HFS–LTP. In contrast, HFS–LTP relies more on adenosine A2 receptors and cAMP-dependent protein kinase A (PKA) compared to TBS–LTP (Zhu et al., 2015). In the present study, heterozygous Q175FDN mice were more susceptible to deficits in HFS–LTP compared to TBS–LTP. At 6–9 months of age, no significant difference was noted between LTP strength in WT and heterozygous Q175FDN mice following TBS. In contrast, HFS–LTP was significantly impaired in heterozygous Q175FDN mice at 6 months of age. While both forms of LTP have been shown to be impaired in HD mice (Usdin et al., 1999; Murphy et al., 2000; Lynch et al., 2007; Simmons et al., 2009; Brito et al., 2014; Kolodziejczyk et al., 2014; Giralt et al., 2017), the earlier onset of the HFS–LTP deficit may be indicative of early adenosine A2 receptor and/or PKA signaling deficits. In R6/1 mice, PKA activity is elevated in the hippocampus, resulting in the hyperphosphorylation of various PKA substrates (Giralt et al., 2011). The adenosine A2 receptor, a G-protein coupled receptor positively linked to adenylate cyclase and PKA, was shown to be transiently elevated at a presymptomatic age in R6/2 mice (Dias et al., 2012). It is worth pointing out that while PKA plays a critical role in HFS–LTP, it seems to have little involvement in HFS–PTP (Lu et al., 2007); in the present study, at 6 months of age, HFS–LTP was impaired but HFS–PTP was perfectly intact in heterozygous Q175FDN mice. These data suggest a potential involvement of PKA dysfunction in the HD-associated long-term plasticity deficit and is of interest for future studies.

## SUMMARY AND CONCLUSION

In all, the current study demonstrates clear increases in basal synaptic strength as well as deficits in short- and long-term synaptic plasticity in the hippocampus of Q175FDN mice. Interestingly, the short-term plasticity impairments did not emerge or progress in parallel with the observed impairments in long-term plasticity, and Q175FDN heterozygous mice were more susceptible to LTP impairment following HFS compared to TBS. Our data suggest that the various plasticity deficits in the HD hippocampus are unlikely to be mediated by a common mechanism; rather, independently developing perturbations in a variety of signaling pathways are likely to combine to contribute to HD hippocampal pathogenesis. As plasticity deficits were exacerbated in homozygous mice that lack non-pathogenic huntingtin, it is of interest for future studies to determine whether allele-selective huntingtin-lowering strategies (Southwell et al., 2018) will prove to be more beneficial than non-selective strategies in preventing or restoring HD-associated plasticity impairments.

## Data Availability

The datasets generated for this study are available on request to the corresponding author.

## Ethics Statement

All experiments with live animals were approved by the Memorial University’s Animal Care Committee in accordance with the guidelines set by the Canadian Council on Animal Care.

## Author Contributions

JQ performed the experiments. MP designed and supervised the experiments. Both authors analyzed the data and wrote the manuscript.

## Conflict of Interest Statement

The authors declare that the research was conducted in the absence of any commercial or financial relationships that could be construed as a potential conflict of interest.
